# Systematic review: effect of whole-body computed tomography on mortality in trauma patients

**DOI:** 10.5249/jivr.v7i2.613

**Published:** 2015-07

**Authors:** Shahab Hajibandeh, Shahin Hajibandeh

**Affiliations:** ^*a*^School of Medicine, University of Liverpool, Liverpool, United Kingdom.

**Keywords:** Whole-body, Computed tomography, Mortality, Trauma

## Abstract

**Background:**

The initial diagnostic evaluation and management of trauma patients is mainly based on Advanced Trauma Life Support (ATLS) guidelines worldwide. Based on ATLS principles, conventional diagnostics such as conventional radiography (CR) and focused abdominal sonography in trauma (FAST) should precede selective use of CT. Whole-body CT (WBCT) is highly accurate and allows detection of life threatening injuries with good sensitivity and specificity. WBCT is faster than conventional diagnostics and saves more time in management of trauma patients. This study aims to review studies investigating the effect of WBCT on mortality in trauma patients.

**Methods:**

Literatures were found by searching keywords in Medline, PubMed and Cochrane library. The relevant articles were selected by two independent reviewers based on title, abstract and introduction sections. Full-texts of selected articles were reviewed and those investigating effect of WBCT on mortality in trauma patients were included.

**Results:**

Searching the keywords in Medline and PubMed resulted in 178 and 167 articles, respectively. Nine studies met the inclusion criteria and were reviewed. These included 8 retrospective and 1 prospective cohort studies. Mortality was measured as mortality rate or standardised mortality ratio (SMR) in the included studies.

**Conclusions:**

Unlike previous systematic reviews, this review indicates that use of WBCT in blunt trauma patients is associated with reduced overall mortality rate and that WBCT can potentially improve the probability of survival in haemodynamically stable and unstable blunt trauma patients. High quality RCTs are required to describe a causal relationship between WBCT and mortality in trauma patients.

## Introduction

Trauma has been one of the leading health problems of the world for a long time. About 5.8 million people die each year as a result of injuries. This accounts for 10% of the world’s deaths.^1^ It is predicted that road traffic accidents will emerge as the fifth leading cause of death in 2030, rising from its position as the ninth leading cause in 2004.^2^

The initial diagnostic evaluation and management of trauma patients is mainly based on Advanced Trauma Life Support (ATLS) guidelines worldwide. The ATLS guidelines include a fast and priority-based physical examination as well as screening radiography supplemented with selective computed tomography (CT). Based on ATLS principles, conventional diagnostics such as conventional radiography (CR) and focused abdominal sonography in trauma (FAST) should precede selective use of CT.^3^

Whole-body CT (WBCT) is highly accurate and allows detection of life threatening injuries with good sensitivity and specificity.^4,5^ It is associated with small number of missed diagnosis which can lead to better patient management.^6,7^ In management of trauma patients, time is very critical. Early detection of life threatening injuries facilitates earlier critical decision-making.^8^ WBCT is faster than conventional diagnostics and saves more time in management of trauma patients.^9-14^ Moreover, having a CT scanner in the trauma room contributes to better time management.^8^

Use of WBCT as initial diagnostic tool in management of trauma patients has been recommended by some authors.^4,7,15,16^ However, despite advantages of WBCT in terms of diagnostic quality and time management, disadvantages such as radiation exposure, costs, and patient transportation to the CT room if the scanner is not located in the trauma room have made it controversial to use WBCT as initial diagnostic tool in management of trauma patients.^17,18^

Knowledge about effect of WBCT on clinical outcomes is essential for determining whether the use of WBCT as initial diagnostic tool in management of trauma patients is justified. Previous systematic reviews on this topic (Sierink JC 2012, van Vugt R 2012 and Healy DA 2013) did not find statistically significant difference in mortality between WBCT and non-WBCT. New studies have been published since these reviews making a new review worthwhile. Therefore, this study aims to review more studies investigating the effect of WBCT on mortality in trauma patients.

## Methods

**Search strategy**

In order to find appropriate articles about the effect of WBCT on mortality in trauma patients Ovid Medline (1946 to October 2013), PubMed and the Cochrane library were used as online databases. Abstracts from trauma associations such as The American Association for the Surgery of Trauma and also Orthopaedic Trauma Association were assessed.

In Medline, the keywords “total body ct” , Mesh term “whole body imaging”, “whole body ct”, “full body ct”, ”tbct”, ”fbct” ,“wbct”,”whole body”, “total body”, “full body” and “pan-ct” were combined by OR (search A). Also, the keywords “ct” , Mesh terms ”tomography, X-ray computed” ,”ct scan” and “scan” were combined by OR as well (search B). On the other hand, the keywords “trauma”, Mesh terms ”wounds and injuries”, “polytrauma” and “multiple trauma” were combined by OR (search C). The resulted literatures from search A, B and C were combined by AND in order to narrow the results.

In PubMed, search strategy consisted of [[“total body ct” OR “whole body ct” OR “full body ct” OR “total body” OR ”whole body” OR “full body”] AND [ “trauma” OR “polytrauma” OR “multiple trauma” OR “injury”] AND [“ct” OR “”computed tomography” OR “ct scan” OR “imaging” OR “scan”] AND [ “mortality” OR “survival”]].

**Study selection**

The title, abstract and introduction sections of the obtained literatures were assessed carefully by two independent reviewers to find relevant articles. After assessing full-text of relevant articles, those articles that met the inclusion criteria of this study were selected to be reviewed. Moreover, in order to reduce the possibility of missing relevant articles, the reference lists of selected articles were reviewed. Any discrepancies in inclusion were resolved by discussion between the reviewers. If necessary, an independent third reviewer was consulted.

**Inclusion criteria**

• Investigate trauma (blunt or penetrating) as condition of interest

• Investigate WBCT as intervention of interest

• Investigate mortality or survival as outcome (In-hospital mortality rate, overall mortality rate, mortality or survival to discharge or standardised mortality ratio)

• Randomised control trials (RCTs) or observational studies (cohort or case-control)

**Exclusion criteria**

• Review articles

• Case reports

• Case series

• Clinical audits

• Ongoing trials

• Authors’ replies

• Language other than English

**Data extraction**

The data from the included articles were extracted on data extraction sheets by two independent reviewers. The extracted data included: publication year, sample size, study design, patient characteristics, type of patients, type of intervention and outcomes. Disagreements were resolved by discussion between the two reviewers. If no agreement could be reached, a third reviewer made the final decision.

**Methodological quality**

The methodological quality of the included articles were assessed by two independent reviewers using SIGN (Scottish Intercollegiate Guidelines Network) notes on methodology checklist^19^ which consists of two sections and classifies each study as high quality, acceptable or low quality. Disagreements were resolved by discussion between the two reviewers. If no agreement could be reached, a third reviewer made the final decision.

**Statistical analysis**

Mortality rate, Trauma and injury-severity score (TRISS)-based SMR and Revised Injury Severity Classification (RISC)-based SMR were outcome measures of this study. Mortality rates between studies were assessed by odds ratio analysis using Stats Direct. For TRISS-based SMR and RISC-based SMR summary analyses of SMR for WBCT and non-WBCT groups were performed.

## Results

**Figure 1:**
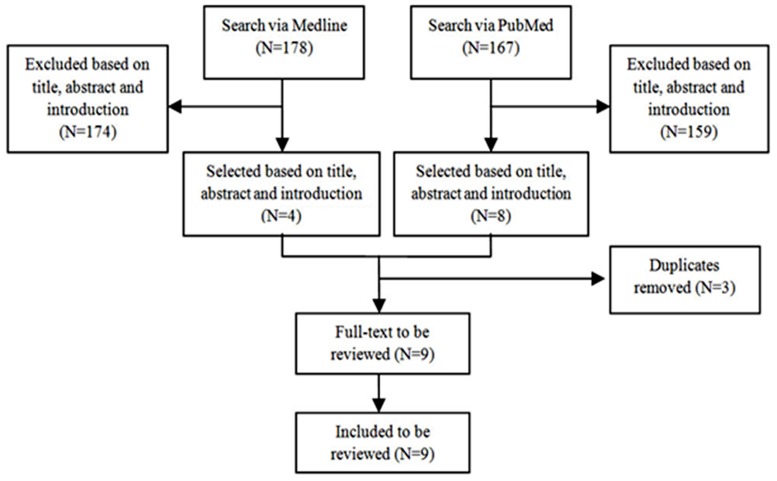
Flow chart for the review

Searching the keywords in Medline and PubMed resulted in 178 and 167 articles, respectively. No relevant article was found in the Cochrane library. Out of these only 9 studies (Huber-Wagner 2013,^20^ Huber-Wagner 2009,^21^ Yeguiayan 2012,^22^ Wada 2013,^23^ Kimura 2013,^24^ Hutter 2011,^25^ Weninger 2007,^26^ Wurmb 2010^27^ and Kanz 2010 ^28^) met the inclusion criteria to be reviewed(Figure 1 and Table 1).

**Table 1:** Main characteristics of included studies.

Study	Design	No of patients	Exposure		Outcome	Methodological Quality*
			Intervention	Control		
**Huber-Wagner (2013)**^20^	Retrospective cohort	16719	WBCT	Non-WBCT	Mortality rate SMR	Acceptable
**Huber-Wagner (2009)**^21^	Retrospective cohort	4621	WBCT	Non-WBCT	Mortality rate SMR	Acceptable
**Yeguiayan (2012)**^22^	Prospective cohort	1950	WBCT	Non-WBCT	Mortality rate	Acceptable
**Wada (2013)**^23^	Retrospective cohort	152	WBCT	Non-WBCT	Mortality rate SMR	Acceptable
**Kimura (2013)**^24^	Retrospective cohort	5208	WBCT	Non-WBCT	Mortality rate SMR	Acceptable
**Hutter (2011)**^25^	Retrospective cohort	313	WBCT	Non-WBCT	Mortality rate	Acceptable
**Weninger (2007)**^26^	Retrospective cohort	370	WBCT	Non-WBCT	Mortality rate	Acceptable
**Wurmb (2010)**^27^	Retrospective cohort	318	WBCT	Non-WBCT	Mortality rate	Acceptable
**Kanz (2010)**^28^	Retrospective cohort	4817	WBCT	Non-WBCT	Mortality rate SMR	Acceptable

SMR: Standardised mortality ratio, WBCT: Whole-body computed tomography,*:Based on SIGN notes on methodology checklist

Huber-Wagner 2013 is a multicentre retrospective study which investigated the effect of WBCT on mortality in haemodynamically unstable trauma patients. This study involved 16719 adult blunt major trauma patients and compared mortality between patients who underwent WBCT during resuscitation and those who did not receive WBCT in three subgroups (severe, moderate and no shock subgroups).^20^

Huber-Wagner 2009 is also another multicentre retrospective study which investigated the effect of WBCT on mortality during trauma resuscitation. This study included 4621 blunt trauma patients and investigated mortality as outcome in WBCT and non-WBCT groups.^21^

Yeguiayan 2012 is a multicenter prospective cohort study that investigated the impact of WBCT on mortality and management of patients with severe blunt trauma. In this study 1950 patients were divided into two groups, WBCT and non-WBCT (selective CT).^22^

Wada 2013 is a retrospective study that investigated impact of WBCT before emergency bleeding control on survival in patients with severe blunt trauma. 152 patients with blunt trauma were divided into 2 subgroups, Trauma and injury-severity score (TRISS) Probability survival (Ps) ≥50% group and TRISS Ps <50% group.^23^

Kimura 2013 is a multicenter retrospective study which investigated the effect of WBCT on mortality in blunt trauma patients with moderate-to-severe consciousness disturbance. It included 5208 patients with systolic blood pressure of greater than 75mmHg and GCS score between 3 and 12.^24^

Hutter 2011 is a retrospective cohort study that investigated association between WBCT policy and survival in blunt major trauma. This study had 2 cohorts. Control cohort included 313 patients who did not undergo a WBCT due to the unavailability of the method. Intervention cohort consisted of two subgroups, patients who were eligible but not scheduled for WBCT (n = 223) and eligible patients who underwent WBCT (n = 608).^25^

Weninger 2007 is a single centre retrospective study which investigated the effect of WBCT on mortality in blunt trauma patients.^26^

Wurmb 2010 is another single centre retrospective study which compared the effect of WBCT on mortality in trauma patients with suspected multiple injuries. It included 318 blunt and penetrating trauma patients.^27^

Kanz 2010 is a multicentre retrospective study that compared probability of survival (Ps) and time management in 4817 major trauma patients who received either WBCT or non-WBCT .^28^

Based on SIGN notes on methodology checklist, the included studies had acceptable methodological quality.

All the included studies considered mortality as outcome. It has been measured either as mortality rate or standardised mortality ratio (SMR). SMR has been calculated based on either TRISS or Revised Injury Severity Classification (RISC) score. Moreover, logistic regression models have been used to analyse association between WBCT and mortality in most of the included studies.

**Mortality rate**

**Figure 2:**
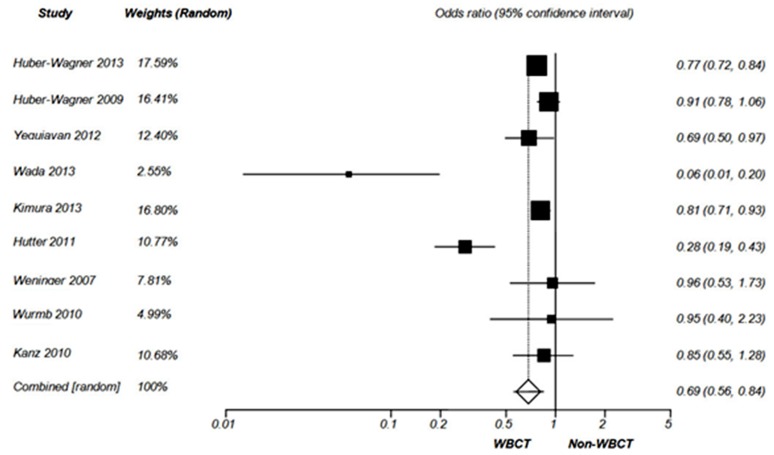
Forest plot of odds ratio for mortality (Random effect).

Mortality rate has been measured by all the included studies (Table 2). Huber-Wagner 2013, Huber-Wagner 2009, Kimura 2013, Hutter 2011 and Kanz 2010 measured overall mortality rate based on survival to discharge. 30-day mortality rate was measured by Yeguiayan 2012 and Wurmb 2010. Wada 2013 and Weninger 2007 measured 28-day mortality rate and in-hospital mortality rate, respectively. The results of odds ratio analysis of mortality are shown by Figure 2.

**Table 2:** Mortality rates reported by the included studies.

		Mortality Rate (95% CI)		
		WBCT	Non-WBCT	Statistical significance
**Huber-Wagner 2013**^20^	**Overall**	17.4%	21.4%	S
	**Severe Shock**	42.1%	54.9%	S
	**Moderate Shock**	18.1%	22.6%	S
	**No Shock**	12.6%	15.6%	S
**Huber-Wagner (2009)**^21^		21%	22%	NS
**Yeguiayan (2012)**^22^		16%	22%	S
**Wada (2013)**^23^		18.1%	80%	S
**Kimura (2013)**^24^		24%	28%	S
**Hutter (2011)**^25^		8%	23%	S
**Weninger (2007)**^26^		17%	16%	NS
**Wurmb (2010)**^27^		8.6%	9.0%	NS
**Kanz (2010)**^28^		18.8%	22.0%	NS

WBCT: Whole-body computed tomography, S:Significant, NS: Not significant , CI: Confidence interval

**TRISS-Based SMR analysis**

**Figure 3:**
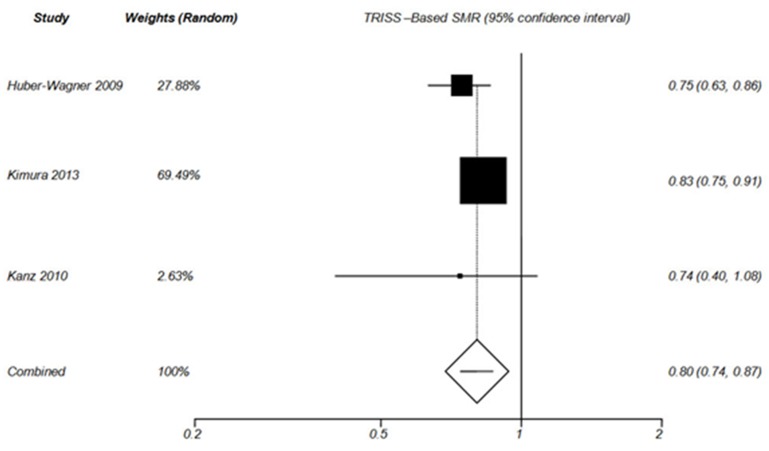
Forest plot of TRISS –Based SMR in WBCT group (Random effect).

**Figure 4:**
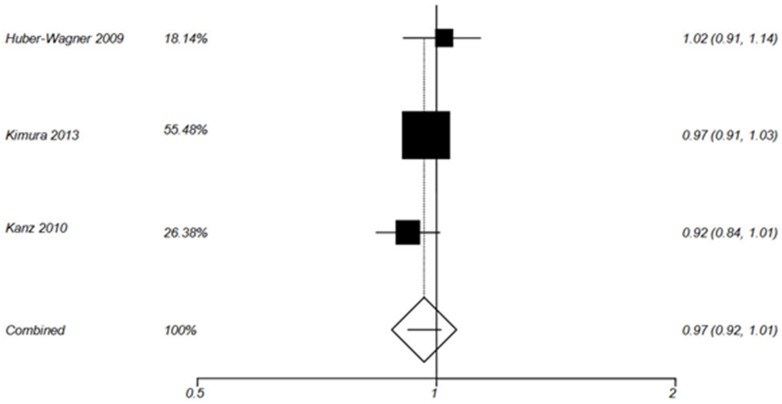
Forest plot of TRISS –Based SMR in non-WBCT group (Random effect).

SMR (defined as ratio of recorded mortality to expected mortality) based on TRISS has been calculated by Huber-Wagner 2009, Wada 2013, Kimura 2013 and Kanz 2010 (Table 3). TRISS-based probability of survival has been measured by Yeguiayan 2012 and Weninger 2007; however, these two studies did not calculate SMR. The results of TRISS-based SMR summery analysis for WBCT and non-WBCT are shown by Figure 3 and Figure 4, respectively.

**Table 3:** SMRs reported by the included studies.

		TRISS –Based SMR (95% CI)		RISC-Based SMR (95% CI)	
		WBCT	Non-WBCT	WBCT	Non-WBCT
**Huber-Wagner (2013)**^20^	**Overall**	-	-	0.85 (0.81–0.89) P=S	0.98 (0.94–1.02) P=S
	**Severe Shock**	-	-	0.99 (0.92–1.06) P=S	1.10 (1.02–1.16) P=S
	**Moderate Shock**	-	-	0.85 (0.78–0.93) P=S	1.03 (0.94–1.12) P=S
	**No Shock**	-	-	0.78 (0.73–0.83) P=S	0.90 (0.84–0.96) P=S
**Huber-Wagner (2009)**^21^		0.745 (0.633–0.859) P=S	1.023 (0.909–1.137 P=NS	0.865 (0.774–0.956) P=S	1.034 (0.959–1.109 P=NS
**Wada (2013)**^23^	TRISS Ps ≥50%	0.63 (0.3-1.0) P=NS	1.40 (-3.07-5.87) P=NS	-	-
	**TRISS Ps <50%**	0.65 (0.41- 0.9) P =S	1.15 (0.98-1.31) P=NS	-	-
**Kimura (2013)**^24^		0.83 (0.75-0.91) P=S	0.97 (0.91-1.03) P=NS	-	-
**Kanz (2010)**^28^		0.74 (0.40-1.08) P=NS	0.92 (0.84-1.01) P=NS	0.69 (0.47-0.92) P=S	0.995 (0.94-1.06) P=NS

SMR: Standardised mortality ratio, WBCT: Whole-body computed tomography, TRISS: Trauma and injury severity score, Ps: probability survival RISC = revised injury severity classification score, S: Significant, NS: Not significant .CI: Confidence interval,

**RISC-Based SMR analysis**

**Figure 5:**
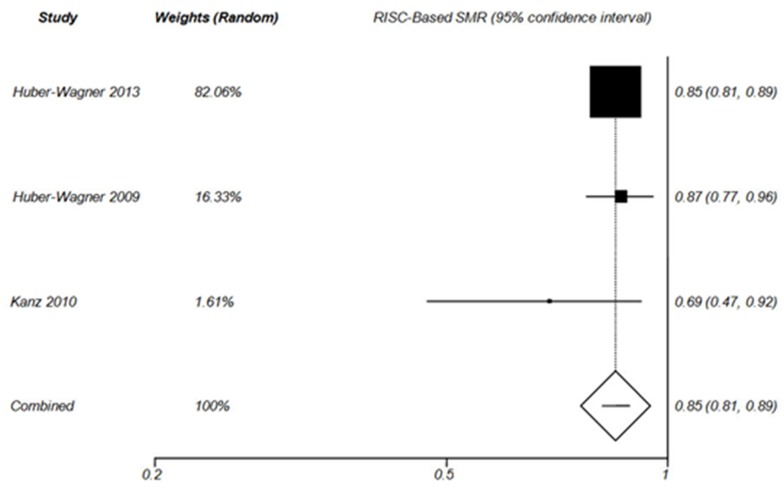
Forest plot of RISC –Based SMR in WBCT group (Random effect).

**Figure 6:**
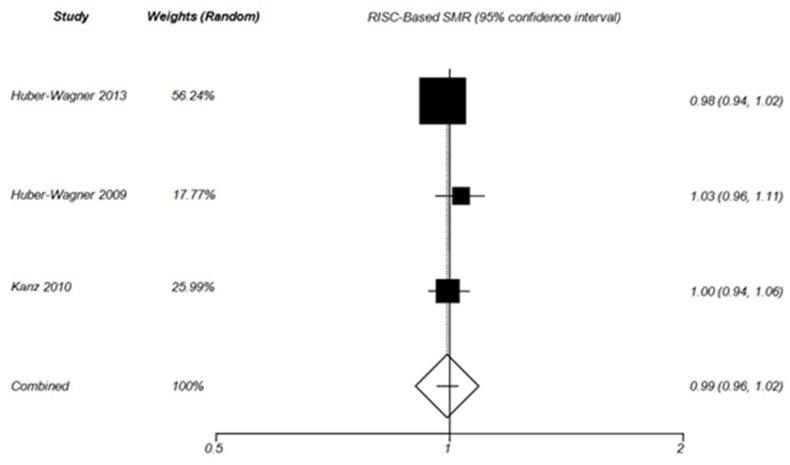
Forest plot of RISC –Based SMR in non-WBCT group (Random effect).

SMR based on RISC score has been measured by Huber-Wagner 2013, Huber-Wagner 2009 and Kanz 2010 (Table 3). The results of RISC-based SMR summery analysis for WBCT and non-WBCT are shown by Figure 5 and Figure 6, respectively.

**Logistic regression analysis**

Huber-Wagner 2013, Huber-Wagner 2009, Yeguiayan 2012, Wada 2013, Kimura 2013 and Hutter 2011 analysed the association between WBCT and mortality by using different logistic regression models.

## Discussion

**Mortality rate**

In the current study, the results of odds ratio analysis showed that use of WBCT in blunt trauma patients is associated with reduced overall mortality [OR= 0.69 (95% CI 0.56-0.84), P= 0.0003] (Figure 2).

Reviewing the included studies showed that there was a significant reduction in overall mortality rate between blunt trauma patients who received WBCT and those patients who did not. Huber-Wagner et al (2013) ^20^ showed that WBCT decreased overall mortality rate in both haemodynamically stable and unstable patients who were in no shock, moderate shock or severe shock. The recorded blood pressure on admission was the only measure to classify patients as haemodynamically stable or unstable in this study. This may subject the results of this study to bias.

According to Wada et al,^23^ WBCT resulted in lower mortality rate in blunt trauma patients who required emergency bleeding control (18.1% vs 80%, P <0.001). When interpreting the results of Wada et al, it should be noted that small sample size in non-WBCT group and significant differences in baseline characteristics between two groups can subject the results to bias. In fact, injury-severity score (ISS), systolic blood pressure, revised trauma score, base excess and lactate levels, number of blood transfusions within 24 hours and number of fresh frozen plasma transfusions within 24 hours were in favour of WBCT group. All of these, together with small sample size, may have caused high mortality rate in non-WBCT group.

Reduction in overall mortality rate was also reported by Yeguiayan et al, , Kimura et al and Hutter et al. Yeguiayan et al ^22^ showed that use of WBCT resulted in lower mortality rate in WBCT group compared to non-WBCT group (16% vs 22%, P=0.02). Moreover, Kimura et al reported that mortality in blunt trauma patients with moderate-to-severe consciousness disturbance who received WBCT was lower than those who did not receive WBCT (24% vs 28%, P=0.0002). ^24^ Hutter et al reported 8% and 23% mortality rate in WBCT and non-WBCT group, respectively (P <0.001).^25^ Considering the fact that all of these studies had observational cohort design, the residual confounding effects cannot be ruled out. Moreover, they showed association between WBCT and reduced mortality; therefore, casual relationship cannot be proven.

Unlike the other studies, Huber-Wagner (2009) and Kanz 2010 studies did not show any statistically significant difference in mortality rate between WBCT and non-WBCT. This is consistent with results of Weninger 2007 and Wurmb 2010 studies. Weninger et al reported similar in-hospital mortality rates in the two groups (17% vs 16%), and Wurmb et al found no significant difference in 30-day mortality rates (8•6 % vs 9•0%).^29^ However, it should be noted that Weninger 2007 and Wurmb 2010 had small sample size and they are subject to bias due to their single centre retrospective design. Also, the number of patients in WBCT group was significantly smaller than non-WBCT group in Kanz 2010.

**TRISS-Based SMR **

According to TRISS –Based SMR analysis in the current study, WBCT increased the probability of survival in blunt trauma patients [Pooled SMR= 0.80 (95% CI 0.74-0.87), P< 0.0001](Figure 3). However, the probability of survival did not improve in non-WBCT patients [Pooled SMR= 0.97 (0.92, 1.01), P = 0.159] (Figure 4). 

Huber-Wagner et al (2009) reported that recorded mortality was lower than mortality predicted by TRISS in patients who received WBCT [SMR= 0.745 (95% CI 0.633-0.859), P<0.001], whereas the probability of survival did not improve in non-WBCT group.^21^ This is consistent with the results reported by Kimura et al [SMR=0.83 (0.75-0.91)].^24^

The results reported by Wada et al showed significant improvement in the probability of survival in patients with TRISS Ps<50% who received WBCT [SMR=0.65 (95% CI 0.41- 0.9), P=0.004]. Moreover, in this study WBCT improved survival of patients who were haemodynamically unstable [SMR= 0.54 (95% CI 0.16-0.91), P = 0.014]. However, WBCT did not improve the probability of survival significantly in patients with TRISS Ps≥50%. This may suggest that WBCT is associated with better survival in patients at high risk of death. ^23^

Unlike the other studies, there was no significant difference in TRISS-based probability of survival in Kanz 2010. This may be due to the fact that TRISS calculation could be performed only in 59.4% of WBCT patients and in 48.2% of non-WBCT patients.^28^ So, TRISS findings are subject to bias in this study.

TRISS is the most widely used method for measurement of expected outcome in patients with trauma. It combines the revised trauma score, which consists of on-the-scene Glasgow coma scale, systolic blood pressure, and respiratory rate, with the discharge diagnoses, age, and mechanism of trauma (blunt vs penetrating) based on the injury-severity score (ISS).^21^ However, it has been argued that the use of the TRISS method is questionable for investigating the effect of WBCT on mortality. In fact, the TRISS equation is based on the ISS, which depends on whether or not patients are given WBCT. The better detection of trauma lesions by WBCT results in increased ISS. This will lead to an increased predicted mortality and thus to bias in conclusions about the benefit of WBCT (Will-Rogers Phenomenon).^22,30^

In Huber-Wagner 2009 and Kimura 2013 studies baseline ISS in WBCT group were significantly greater than non-WBCT group. This is associated with overestimation of TRISS predicted mortality in WBCT group and thus can potentially bias the results.

**RISC-Based SMR**

The results of RISC-Based SMR analysis were consistent with the results of TRISS–Based SMR analysis. In fact, the probability of survival improved in WBCT patients (Figure 5) but not in Non-WBCT patients (Figure 6). Huber-Wagner et al (2013), Huber-Wagner et al (2009) and Kanz et al^28^ reported that WBCT improved the probability of survival based on RISC score. The RISC score is one of the most precise trauma outcome prediction models. It is calculated on the basis of more variables compared to TRISS. ^20^

Although baseline ISS between WBCT and non-WBCT groups were different in Huber-Wagner 2013 study, authors argued that slightly higher ISS based on the diagnoses obtained in the WBCT group is not responsible for the increased probability of survival in this group and the results are not subject to Will-Rogers Phenomenon.^20^

The logistic regression analysis in the included studies^20-25^ showed statistically significant reduction in the mortality risk among WBCT patients. This may suggest that WBCT can potentially increase the chance of survival in trauma patients. However, considering the heterogeneity between the included studies and retrospective nature of most of them, the causal relationship can not be proven.

Most of the included studies did not have information about CT protocols and indications for WBCT used in participating trauma centres. So, possible variations in CT protocols may bias the results of these studies. 

**Limitations**

This review has some limitations. None of the included articles was randomised control trial (RCT), which is the gold standard study design for the purpose of this study. The reviewed studies were mainly retrospective observational studies that are subject to bias. Moreover, these studies showed association rather than causal relationship between use of WBCT and mortality. Based on SIGN notes on methodology checklist the included studies were acceptable in terms of methodological quality. However, high quality studies are essential for making a robust conclusion. There were some variations between baseline characteristics, included populations and study designs between the reviewed articles. Also, outcome definition and mortality analysis methods were not identical in all the included studies. This made it impossible to directly compare the results of all studies together and can potentially subject the results of this review to bias.

In future systematic reviews, these limitations can be avoided when more RCTs with more comparable population and outcomes are published.

## Conclusion

Unlike previous systematic reviews, this review indicates that use of WBCT in blunt trauma patients is associated with reduced overall mortality rate compared to conventional imaging. Moreover, WBCT can potentially improve the probability of survival in both haemodynamically stable and unstable blunt trauma patients.

This review suggests association between WBCT and reduced mortality in trauma patients; however, considering the heterogeneity between the current studies in terms of included population and outcome definitions, high quality RCTs with (24-h or in-hospital) mortality as outcome of interest are required to provide more robust evidences about use of WBCT in trauma patients and to describe a causal relationship between WBCT and mortality in trauma patients.

The REACT-2 trial (http://ClinicalTrials.gov/ NCT01523626) is an ongoing international multicenter RCT that compares immediate WBCT during the primary survey of severely injured trauma patients with conventional imaging strategies. The results of this trial will provide better evidence about effect of WBCT on mortality.
